# Incidence of necrotising enterocolitis before and after introducing routine prophylactic *Lactobacillus* and *Bifidobacterium* probiotics

**DOI:** 10.1136/archdischild-2019-317346

**Published:** 2019-10-30

**Authors:** Claire Robertson, George M Savva, Raducu Clapuci, Jacqueline Jones, Hassan Maimouni, Eleanor Brown, Ashish Minocha, Lindsay J Hall, Paul Clarke

**Affiliations:** 1 Neonatal Unit, Norfolk and Norwich University Hospitals NHS Foundation Trust, Norwich, UK; 2 Gut Microbes and Health, Quadram Institute Bioscience, Norwich, UK; 3 Core Science Resources, Quadram Institute Bioscience, Norwich, UK; 4 Norwich Medical School, University of East Anglia, Norwich, UK; 5 Paediatric and Neonatal Surgery, Jenny Lind Children's Hospital, Norfolk and Norwich University Hospitals NHS Foundation Trust, Norwich, UK

**Keywords:** Late-onset sepsis, microbiota, necrotizing enterocolitis, preterm, very low birth weight

## Abstract

**Objective:**

To compare rates of necrotising enterocolitis (NEC), late-onset sepsis, and mortality in 5-year epochs before and after implementation of routine daily multistrain probiotics administration in high-risk neonates.

**Design:**

Single-centre retrospective observational study over the 10-year period from 1 January 2008 to 31 December 2017.

**Setting:**

Level 3 neonatal intensive care unit (NICU) of the Norfolk and Norwich University Hospital, UK.

**Patients:**

Preterm neonates at high risk of NEC: admitted to NICU within 3 days of birth at <32 weeks’ gestation or at 32–36 weeks’ gestation and of birth weight <1500 g.

**Intervention:**

Prior to 1 January 2013 probiotics were not used. Thereafter, dual-species *Lactobacillus acidophilus* and *Bifidobacterium bifidum* combination probiotics were routinely administered daily to high-risk neonates; from April 2016 triple-species probiotics (*L. acidophilus, B. bifidum,* and *B. longum* subspecies *infantis*) were used.

**Main outcome measures:**

Incidence of NEC (modified Bell’s stage 2a or greater), late-onset sepsis, and mortality.

**Results:**

Rates of NEC fell from 7.5% (35/469 neonates) in the pre-implementation epoch to 3.1% (16/513 neonates) in the routine probiotics epoch (adjusted sub-hazard ratio=0.44, 95% CI 0.23 to 0.85, p=0.014). The more than halving of NEC rates after probiotics introduction was independent of any measured covariates, including breast milk feeding rates. Cases of late-onset sepsis fell from 106/469 (22.6%) to 59/513 (11.5%) (p<0.0001), and there was no episode of sepsis due to *Lactobacillus* or *Bifidobacterium*. All-cause mortality also fell in the routine probiotics epoch, from 67/469 (14.3%) to 47/513 (9.2%), although this was not statistically significant after multivariable adjustment (adjusted sub-hazard ratio=0.74, 95% CI 0.49 to 1.12, p=0.155).

**Conclusions:**

Administration of multispecies *Lactobacillus* and *Bifidobacterium* probiotics has been associated with a significantly decreased risk of NEC and late-onset sepsis in our neonatal unit, and no safety issues. Our data are consistent with routine use of *Lactobacillus* and *Bifidobacterium* combination probiotics having a beneficial effect on NEC prevention in very preterm neonates.

**What is already known on this topic?**

- Necrotising enterocolitis (NEC) remains a leading cause of mortality and morbidity in premature and very low birthweight infants.
- The aetiology of NEC is multifactorial, but the development of an abnormal gut microbiota is an important predisposing risk factor.
- Multiple meta-analyses and observational studies have shown that ameliorating gut bacterial colonisation through the enteral administration of live probiotic bacteria significantly reduces NEC incidence.

**What this study adds?**

- Routine daily dosing with *Lactobacillus* and *Bifidobacterium* probiotics was a cheap, simple intervention associated with significantly decreased NEC, surgical NEC and late-onset sepsis in our neonatal unit.
- No episode of sepsis due to *Lactobacillus* or *Bifidobacterium* occurred in the routine probiotic supplementation epoch.
- The apparent impact of reduced NEC incidence with probiotics was particularly pronounced in the first 2 weeks postnatal, implying that achieving early probiotic bacterial gut colonisation is crucial.

## Introduction

Necrotising enterocolitis (NEC) is a leading cause of mortality and morbidity in premature very low birthweight (VLBW; <1500 g) babies.^1 2^ Prevention of NEC is a top UK research priority.^3^ NEC aetiology is multifactorial,^4^ with abnormal gut microbiota increasingly recognised as central.^5^ Probiotics are ‘*live microorganisms that, when administered in adequate amounts, confer a health benefit on the host’*.^6^ Multiple randomised controlled trials (RCTs), systematic reviews and meta-analyses of RCTs and observational studies have shown that prophylactic probiotics prevent NEC in preterm babies.^7–9^ A 2012 meta-analysis,^10^ updated in 2017 to include 25 RCTs and >7000 neonates,^11^ showed strong evidence for using multispecies probiotics to reduce NEC incidence (pooled OR=0.36, 95% CI 0.24 to 0.53, p<0.00001), and associated mortality (OR=0.58, 95% CI 0.43 to 0.79, p=0.0006). Yet at present most UK centres do not offer probiotics routinely.^12^ Uncertainties about effectiveness may arise from RCTs which have not shown benefit, heterogeneity, multiplicity, and dosage of probiotic microorganisms, differing inclusion criteria, treatment durations and NEC definitions used to assess benefit.^13^

There are no published reports on the safety or efficacy of routine probiotics used in any UK centres. We therefore aimed to assess whether routine probiotic supplementation has been associated with a decreased incidence of NEC, sepsis, and all-cause mortality in our centre.

## Methods

Single-centre retrospective review of neonates admitted to our tertiary-level neonatal intensive care unit (NICU) which caters to ~6000 deliveries annually and is a regional neonatal surgical centre.

### Eligible population

‘Eligible high-risk neonates’ comprised all those inborn at <32 weeks’ gestation, plus 32–36 weeks’ gestation VLBW infants. Outborn babies were included if transferred in within 72 hours of birth, providing no abdominal concerns at referral.

### Study period

Eligible neonates were reviewed in two consecutive 5-year epochs, allocated by date of birth: 1 January 2008 to 31 December 2012 (pre-probiotics epoch) and 1 January 2013 to 31 December 2017 (routine probiotic epoch).

### Enteral feeding practices

Our unit adopted a standardised regional enteral feeding guideline for preterm neonates in January 2011. Ideally, human milk feeds commenced on day 1. From May 2013, donor breast milk (DBM) was available to supplement shortfalls in mother’s own breast milk supply before full feeds. Cow’s milk-based fortifier was added to breast milk between full enteral feeds (≥150 mL/kg/day) and discharge—a consistent policy across epochs. Milk type used in the period until full feeds was categorised as exclusive maternal breast milk, exclusive DBM, formula, or mixed (ie, any combination of own maternal milk, DBM, and formula).

### Probiotic exposure and compliance

We introduced routine daily combination *Lactobacillus* and *Bifidobacterium* probiotics for prophylaxis of NEC in high-risk babies resident in our NICU on 1 January 2013, and for all eligible infants subsequently admitted.^14^ Written parental information was provided.^15^ We administered first probiotic dose with first colostrum feeds, ideally on postnatal day 1. Probiotics continued until ~34 weeks postmenstrual age. We initially used the dual-species probiotic Infloran capsules (Desma Healthcare, Chiasso, Switzerland), containing *Lactobacillus acidophilus* and *Bifidobacterium bifidum*, half a capsule (125 mg) twice daily (1×10^9^ colony-forming units (CFU) of each bacterial species daily). From April 2016, we used triple-species Labinic Drops (Biofloratech, Walton-on-Thames, UK), four drops once daily (~0.5×10^9^ CFU dosage each of *L. acidophilus, B*. *bifidum* and *B*. *longum* subspecies *infantis* daily). Probiotic batches were subject to quality control (LJH’s laboratory) for species and CFU confirmation alongside our prospective microbiota study.^16^ Probiotics were prescribed on drug charts in accordance with our NICU’s guideline, and compliance was good.^17^

### Oxygen saturation limits

Between 2008 and May 2011, we targeted oxygen saturation (SaO_2_) range 84%–92% for babies aged <32 weeks. From 31 May 2011, with emergent evidence associating lower SaO_2_ ranges with mortality and NEC,^18^ we targeted 91%–95% SaO_2_.

### Identification and classification of NEC cases

From BadgerNet electronic patient records (CleverMed, UK), we identified all cases of definite, suspected, and perforated NEC. All eligible infants were screened between admission and final discharge home (or death if earlier) from either our NICU/paediatric ward, or step-down NICU/other hospital ward if transferred out. Electronic clinical records, including X-ray request forms, abdominal radiographs, radiologist reports, blood results, surgical notes, histopathology and autopsy reports, and death certificates were independently reviewed by two unblinded clinicians (CR, PC) to determine whether they met NEC case definition according to each of three diagnostic systems: modified Bell’s staging criteria,^19^ Vermont-Oxford Network,^20^ and Battersby *et al*.^1^ We defined ‘surgical NEC’ as any baby with definite NEC in whom an abdominal drain was inserted or who underwent laparotomy. Potential surgical NEC cases whose operative findings and/or histopathology reports were uncertain or required expert interpretation, and also those with uncertainty regarding staging, were additionally reviewed and adjudicated by a consultant paediatric surgeon (AM) blinded to epoch.

We imputed as either NEC Bell’s stage 2b/3a cases babies who had acute clinical deterioration with Bell’s stage 2 or 3 systemic and gastrointestinal signs (eg, blood in stool, abdominal distension, tenderness, acidosis), but without classic stage 2/3 radiographic findings of pneumatosis intestinalis, portal venous gas or pneumoperitoneum, provided treated clinically as presumed NEC with ≥10 days’ antibiotics and nil enterally and with fixed distended loops, definite ascites or gasless abdomen on abdominal X-ray and no alternative diagnosis (eg, sepsis) for the episode. These included babies who underwent surgical drain placement who died before planned laparotomy without autopsy, but with NEC recorded on their death certificate.

Isolated pneumoperitoneum without other clinical/radiographic evidence of NEC was diagnosed as spontaneous intestinal perforation, not NEC. Similarly, any case who satisfied any staging/clinical-radiological definition of NEC but who at proximate surgery or postmortem examination for that episode had no NEC findings was excluded as NEC.

### Identification and classification of late-onset sepsis

Late-onset sepsis was defined as clinical signs of sepsis and a concomitant positive blood or cerebrospinal fluid culture occurring >72 hours after birth and before 46 weeks’ postmenstrual age or discharge/transfer from our NICU if sooner. We reviewed all positive blood and CSF cultures from our local microbiology laboratory database for the whole study period. Repeated growth of the same isolate in successive samples obtained within 7 days was considered the same episode.

### Outcome measures

NEC was coded as a binary outcome, and NEC case definition used for primary analyses was modified Bell’s criteria ≥2a.^19^ Mortality from any cause was included as a secondary outcome.

### Statistical analysis

Full details of statistical methods are provided in online supplementary figure S1. In short, time-to-event analysis was used to estimate the difference in NEC rate between epochs, controlling for potentially confounding variables, and with death and discharge considered competing risks. Cumulative NEC incidence is reported, with sub-hazard ratios (sub-HR: ratio of cumulative incidence rates) reflecting the association between each factor and cumulative incidence.

10.1136/archdischild-2019-317346.supp1 Supplementary data

Effect of epoch appeared to vary significantly over the risk period, thus we conducted a second analysis including three separate coefficients for the effect of epoch on NEC in the first, second and beyond the second week after birth. A regression discontinuity analysis^21^ assessed whether the effect of epoch would be better explained by a linear trend over time or by a step change coinciding with introduction of probiotics. A time-to-event analysis estimated the effect of epoch on all-cause mortality. We used χ^2^ and Fisher’s exact tests to compare mortality rates following NEC diagnosis and other between-epoch proportions.

## Results

### Descriptive analysis

The sample included 982 eligible neonates: 469 born in the pre-probiotics epoch and 513 in the routine probiotics epoch (online supplementary figure S2, patient flow). Table 1 shows comparative distributions of covariates between epochs. The only covariate to differ substantially between epochs was mode of milk feeding in the period up to full feeds: exclusive breast milk feeding was more common in the routine probiotics epoch (table 1).

10.1136/archdischild-2019-317346.supp2 Supplementary data

**Table 1:** NEC incidence and severity, sepsis incidence, and baseline data in the pre-probiotics vs routine probiotics epochs

Factor	Pre-probiotics epoch n=469	Routine probiotics epoch n=513
NEC diagnostic system		
Modified Bell’s ≥2a	35 (7.5%)	16 (3.1%)
Vermont-Oxford	30 (6.4%)	15 (2.9%)
Battersby *et al*	37 (7.9%)	19 (3.7%)
NEC severity		
Modified Bell’s 2a or 2b	6 (1.3%)	2 (0.4%)
Modified Bell’s 3a or 3b	29 (6.2%)	14 (2.7%)
Surgical NEC	30 (6.4%)	15 (2.9%)
Drains only	4	1
Laparotomy±drain	20	13
Died before laparotomy	6	1
Postnatal age at NEC diagnosis (days)	12 (6–21)	18 (11–30)
Postmenstrual age at NEC diagnosis (weeks)	27 (26–29)	28 (26–31)
Mortality before discharge among NEC cases	16 (46%)	3 (19%)
Spontaneous intestinal perforation	2 (0.4%)	6 (1.1%)
Sepsis		
Early onset sepsis	10 (1.1%)	1 (0.2%)
Late-onset sepsis	106 (22.6%)	59 (11.5%)
Responsible isolates*		
CoNS	87 (18.6%)	47 (9.2%)
Gram-negative	19 (4.1%)	6 (1.2%)
Enterococcus	11 (2.3%)	2 (0.4%)
Other organism	15 (3.2%)	6 (1.2%)
*Lactobacillus* or *Bifidobacterium* sepsis	0 (0%)	0 (0%)
Sex, female	225 (48%)	261 (51%)
Mode of delivery		
Vaginal	193 (42%)	229 (45%)
Caesarean	271 (58%)	284 (55%)
PROM		
No	306 (65%)	347 (68%)
Yes	103 (22%)	118 (23%)
Not recorded	60 (13%)	48 (9%)
NSAID treatment for PDA		
None	421 (90%)	487 (95%)
Indometacin	37 (8%)	0 (0%)
Ibuprofen	11 (2%)	26 (5%)
Birth weight (g)	1100 (810–1410)	1100 (832–1430)
Gestational age (weeks)	28 (26–30)	28 (26–31)
SGA	114 (24%)	132 (26%)
Antenatal steroids	405 (87%)	447 (88%)
Received initial antibiotics†	502/599 (83.8%)	509/597 (85.6%)
Days to first enteral feed	2 (1–4)	2 (2–3)
Enteral feeding‡
Never fed	34 (7%)	32 (6%)
Mother’s own breast milk only	198 (42%)	322 (63%)
Donor breast milk only	0 (0%)	9 (2%)
Preterm formula only	50 (11%)	23 (4%)
Mixed (formula and any breast milk)	186 (40%)	127 (25%)

Data are median (IQR) or number (%).

*For individual infants who had at least one such sepsis episode.

†Data presented are for all babies born at <32 weeks’ gestation within the epochs who received initial empirical antibiotics (benzylpenicillin and gentamicin) following admission to NICU.

‡Mode of milk feeding between first feed and full feeds or NEC/death if earlier.

PDA, patent ductus arteriosus; CoNS, coagulase-negative staphylococci;NEC, necrotising enterocolitis; NICU, neonatal intensive care unit; NSAID, non-steroidal anti-inflammatory drug; PROM, prolonged rupture of membranes;SGA, small for gestational age.

### NEC incidence according to diagnostic system used

Table 1 shows NEC incidence in the pre-probiotics and routine probiotics epochs using the three NEC classifications. NEC Bell’s stage ≥2a occurred in 35 (7.5%) babies in the pre-probiotics epoch and 16 (3.1%) in the routine probiotics epoch. Imputed cases (without classical X-ray signs) were n=5 in the pre-probiotics epoch and n=1 in the routine probiotics epoch. In the latter era, three babies who developed NEC during the second postnatal week had not received prior probiotics; dosing had been overlooked in two cases. NEC rates within the routine probiotics epoch were similar irrespective of product used (10 cases during 39 months using Infloran; 6 cases during 21 months using Labinic). More NEC cases of Bell’s stage ≥3a occurred in the pre-probiotics era, 29 (6.2%) vs 14 (2.7%) (p=0.017), but comprised a similar proportion of overall NEC cases within epochs, 29/35 (83%) vs 14/16 (88%) (p=0.70). Surgical NEC occurred in 30 infants in the pre-probiotics epoch (6.4% of eligible neonates) vs 15 (2.9%) in the routine probiotics epoch (p=0.014).

### Cumulative incidence of NEC from birth

**Figure 1:**
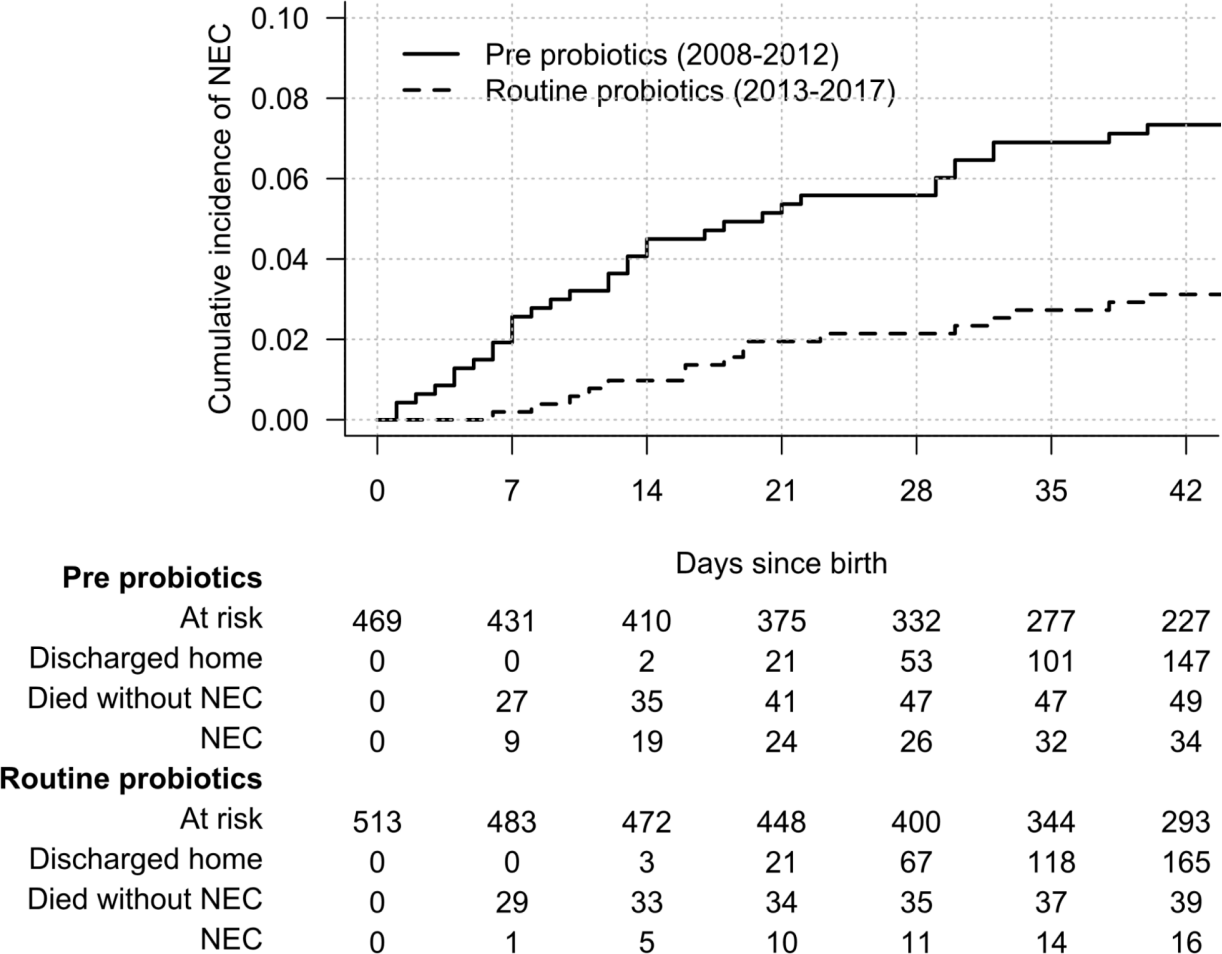
The cumulative incidence of necrotising enterocolitis (NEC) from date of birth stratified by epoch. Risk table shows the number of cases still at risk with each passing week from birth, as well as the cumulative numbers of those diagnosed with NEC, who died without a diagnosis of NEC, or who were discharged home without a diagnosis of NEC. One NEC case was censored in this time-to-event analysis, a baby born in the month before 1 January 2013 who developed NEC after that date, leaving 34 cases in the pre-probiotics epoch and 16 cases in the routine probiotics epoch included.

**Figure 2:**
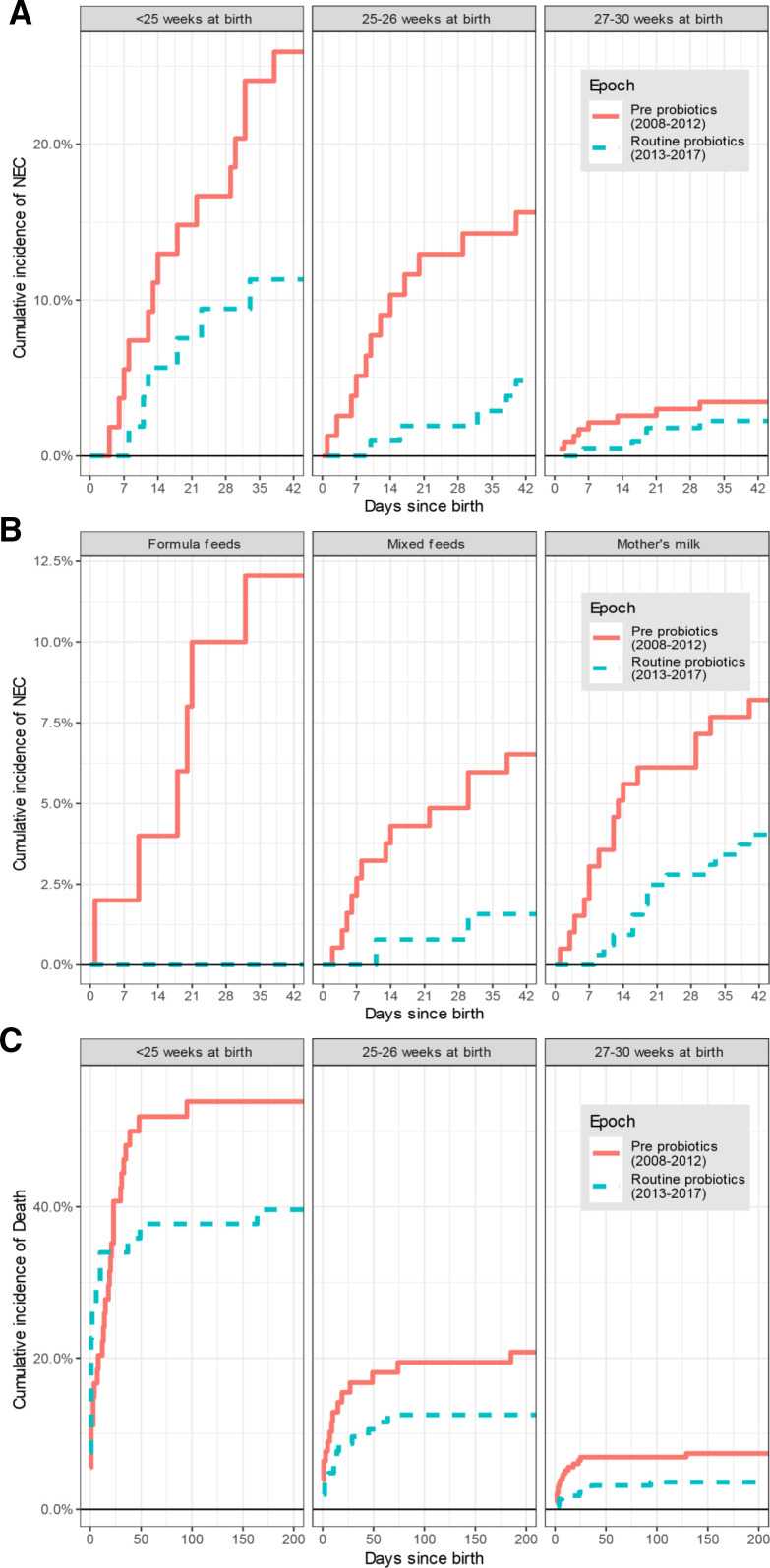
(A) Cumulative incidence of necrotising enterocolitis (NEC) stratified by gestational age at birth and by epoch. (B) Cumulative incidence of NEC stratified by gestational age at birth and by milk type for those who initiated enteral feeds. There was no case of NEC among those fed donor breast milk exclusively and so this group is not shown. (C) Cumulative incidence of mortality up to 200 days after birth, stratified by epoch and gestational age.

Figure 1 shows the cumulative incidence of NEC from day of birth until final discharge home (or earlier death). NEC incidence was strongly associated with birth gestational age (GA) (figure 2). Irrespective of GA, all cases of NEC occurred within 40 days of birth. NEC rate was lower in the routine probiotic-use era for all GA subgroups, with differences particularly pronounced in the first 2 weeks after birth.

The overall sub-HR for being born in the routine probiotics compared with pre-probiotics epoch on NEC rate was 0.43 (95% CI 0.23 to 0.77, p=0.005). When all other covariates were included in the model (table 2), the estimated association between epoch and NEC rate remained very similar (sub-HR=0.44, 95% CI 0.23 to 0.85, p=0.014). However, as reflected in the cumulative incidence curve, the difference between epochs was greatest in the first week after birth, and there was no difference after the second week (test for proportional hazard vs difference in sub-HR over time since birth: p=0.013). Estimates for the association between NEC and probiotics stratified by time since birth are shown in online supplementary table S3. In the first week after birth, the sub-HR for routine versus no probiotics was 0.08 (95% CI 0.01 to 0.59, p=0.014), in the second week was 0.43 (95% CI 0.13 to 1.4, p=0.17) and thereafter was 0.85 (95% CI 0.36 to 2.0, p=0.72).

10.1136/archdischild-2019-317346.supp3 Supplementary data

**Table 2:** Multivariable regression showing the relative risk of NEC associated with each factor

Factor	Level	Sub-HR (95% CI)	P value
Epoch	Routine vs pre-probiotics	0.44* (0.23 to 0.85)	0.014
Gestational age at birth	<25 weeks	1.00 (ref)	
	25–26 weeks	0.34** (0.16 to 0.70)	0.003
	27–30 weeks	0.09*** (0.03 to 0.26)	<0.001
	≥31 weeks	–	–
Milk type†	Mother's milk	1.00 (ref)	
	No enteral feed	0.15** (0.04 to 0.52)	0.003
	Donor milk	–	–
	Formula feed	0.85 (0.29 to 2.52)	0.775
	Mixed	0.82 (0.41 to 1.64)	0.576
Birth weight	<1000 g	1.00 (ref)	
	1000–1499 g	0.71 (0.26 to 1.99)	0.520
	≥1500 g	1.12 (0.22 to 5.84)	0.891
Sex	Male (vs female)	1.05 (0.59 to 1.86)	0.872
Antenatal steroids	At least one dose (vs never)	1.10 (0.45 to 2.68)	0.840
NSAID	None	1.00 (ref)	
	Indometacin	1.21 (0.47 to 3.08)	0.694
	Ibuprofen	1.20 (0.41 to 3.56)	0.738
PROM	No	1.00 (ref)	
	Yes	0.53 (0.22 to 1.29)	0.164
	Not recorded	0.78 (0.33 to 1.88)	0.583
Mode of birth	Caesarean (vs vaginal)	1.82 (0.92 to 3.60)	0.086

There were no cases of NEC among those fed using donor milk, or those born after 31 weeks in either epoch and so these groups are excluded from regression analysis.

*P<0.05, **p<0.01, ***p<0.001,

†Mode of milk feeding between first feed and full feeds or NEC/death if earlier.

NEC, necrotising enterocolitis; NSAID, non-steroidal anti-inflammatory drug; PROM, premature rupture of membranes; Ref., reference group; Sub-HR, sub-hazard ratio.

NEC was also independently linked to GA, and appeared less common before initiation of enteral feeds. There was no evidence that birth weight (once adjusted for GA) or milk type used for feeding were significantly associated with NEC rates, although our study was not designed to test these associations.

### All-cause mortality and NEC-associated rate of death within epochs

There were 67 deaths from all causes before discharge in the pre-probiotics epoch (14.3% of eligible neonates) and 47 in the routine probiotics epoch (9.2%). The cumulative incidence of death among eligible included babies is shown in figure 2, stratified by epoch and GA. All-cause mortality reduced between epochs (sub-HR=0.62, 95% CI 0.42 to 0.90, p=0.013). This was attenuated and no longer statistically significant after adjusting for all covariates (online supplementary table S4). However, as with NEC, the proportional hazard assumption was not met and on stratifying by time since birth there was a significantly lower death rate beyond 2 weeks during the routine probiotics epoch compared with in the pre-probiotics epoch (sub-HR=0.34, 95% CI 0.17 to 0.68, p=0.003; online supplementary table S5). Other factors independently associated with mortality were lower GA, never having received any enteral milk feeds, and non-reception of antenatal steroids.

10.1136/archdischild-2019-317346.supp4 Supplementary data

10.1136/archdischild-2019-317346.supp5 Supplementary data

Death before discharge among diagnosed NEC cases occurred in 15/35 (44%) in the pre-probiotics epoch, compared with 3/16 (19%) in the routine probiotics epoch, p=0.15.

### Regression discontinuity analysis

**Figure 3:**
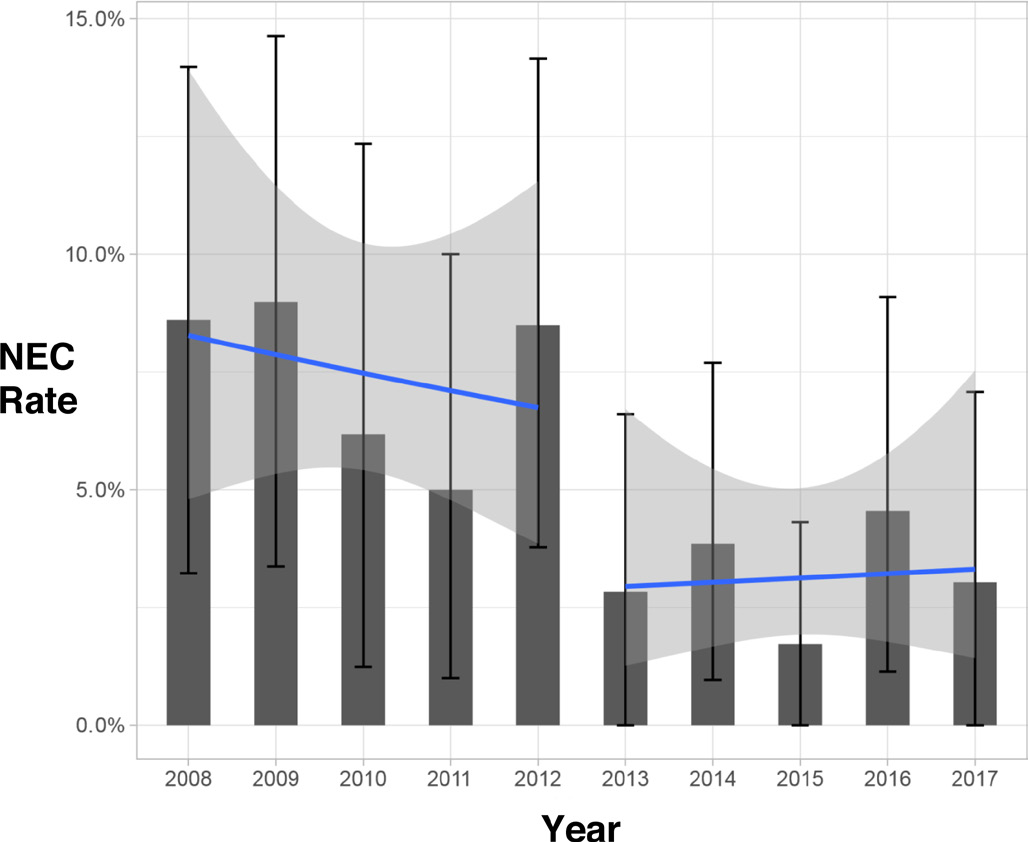
The rate of necrotising enterocolitis (NEC) in each year (solid bars with error bars), and linear trends estimated within each era (lines with error bands). Each estimate is shown with a 95% bootstrapped confidence interval.

Within individual years across the whole study period, the proportion of NEC cases as a proportion of all eligible admissions is shown in figure 3, along with estimates of the trend across years within each epoch. After adjusting for calendar year as a continuous variable in the time-to-event analysis, the effect of epoch was largely unchanged and remained statistically significant in the first week after birth (online supplementary table S3). This suggests that our results are not explained by an underlying linear improvement in NEC rates with time, but rather reflect a step change in incidence around the introduction of routine probiotics administration. Conversely, the improvement in death rate between epochs is better explained by a linear trend over time (online supplementary table S5).

### Sensitivity analyses

Estimates were similar when postmenstrual age was used as a time-scale instead of days since birth (data not shown). Since milk type used between first and full feeds was the only other recorded variable to change significantly between epochs, we also stratified plots by mode of milk feed (among those who initiated enteral feeds) (figure 2); this showed a clear reduction in NEC rate in the routine probiotics epoch irrespective of milk feed type. Replacing ‘birth weight’ with ‘small for gestational age’ in the multivariable analysis did not affect findings. Repeating analyses using the Vermont-Oxford Network NEC case definition^20^ did not significantly affect findings either: the estimate of effect of epoch was sub-HR=0.47 (95% CI 0.24 to 0.92, p=0.027), and the stratified analysis was also largely consistent. The only difference was in the regression discontinuity analysis, where the evidence was less strong that the trend was due to probiotics introduction as opposed to a linear trend (in the first week after birth, adjusted sub-HR=0.09, 95% CI 0.00 to 1.62, p=0.10), although this involved a very small number and was still consistent with a big drop in NEC rate in the first week after birth.

### Incidence of late-onset sepsis

Babies having one or more episodes of late-onset sepsis were significantly fewer in the routine probiotics epoch compared with the previous epoch (p<0.0001), and there were fewer episodes of coagulase-negative staphylococcal sepsis (p<0.0001) (table 1).

No case of any probiotic-organism bacterial sepsis was observed in either epoch.

## Discussion

### Summary of findings

In this sample of 982 high-risk neonates admitted to our NICU over a 10-year period, NEC rate fell by more than half (from 7.5% to 3.1%) in the 5-year period following the introduction of a policy of routine multispecies probiotics administration from birth (~55% relative risk reduction). Our apparent more-than halving of NEC risk with probiotics mirrors remarkably the highly significant risk reductions shown in large meta-analyses of RCTs and observational studies,^7–11^ including that of a very large German observational study which also used Infloran.^22^ While some quality improvements occurred in our NICU over the study period, the difference observed was not attributable to changes in the main important covariates assessed between epochs, including exposure to breast milk. The improvement affected all high-risk neonates irrespective of GA. The impact appeared greatest in the first 2 weeks after birth, suggesting that aiming for very early postnatal probiotic bacterial gut colonisation is crucial. Late-onset sepsis rates also halved in the routine probiotics epoch compared with in the previous epoch (from 22.6% to 11.5%). There was a fall in the all-cause mortality rate between epochs, although this was more consistent with a continuous trend over the study period.

### Strengths and limitations

This is the first UK study to evaluate the potential impact of routine probiotics use on NEC rates. We used multispecies *Lactobacillus* and *Bifidobacterium* probiotics, shown to be most effective for NEC prophylaxis in large meta-analysis.^11^ These were routinely available, and commercially produced to the same accredited Good Manufacturing Practice standards required for drug production. We had a high rate of compliance and early probiotics administration in eligible babies. We applied three different NEC classification systems; all showed similar reductions in NEC rates across epochs. A further study strength is the variety of rigorous statistical analyses applied.

Being retrospective and observational, our findings only support an association and cannot conclusively determine that probiotics caused the observed markedly reduced NEC incidence; unmeasured confounders might underlie observed differences between epochs. The only measured covariate observed to change between epochs was milk-type used, but this did not explain the improved NEC rate. Furthermore, a regression discontinuity analysis suggested that the drop in NEC rate was better explained by a step-change at the introduction of probiotics, and no other significant policy changes occurred between epochs that could have affected NEC incidence so markedly.

Importantly, colonisation and development of the early life microbiota, including *Bifidobacterium* and *Lactobacillus* species, occurs immediately postbirth. Previous studies (clinical and preclinical) indicate that optimal colonisation by supplemented species in a ‘naïve’ gut environment enables more effective persistence, linking to improved short-term and long-term host health.^23–25^

Choice of bacterial species and strains is a crucial consideration. Many previous studies already highlighted that strains of species *B*. *bifidum*, *B*. *longum* subspecies *infantis,* and *L*. *acidophilus* were efficacious in other NICUs globally, key to our selecting these combinations for routine use.

Mechanistically, *B*. *bifidum* and *B*. *infantis* strains are pioneer colonisers of the infant gut, and their innate ability to digest components of breast milk preferentially, for example, human milk oligosaccharides (HMOs), augments their establishment within the infant gut.^26 27^ Numerous studies have shown how these species may provide resistance to potentially pathogenic microbes, so-called ‘colonisation resistance’, and also aid development of the mucosal and systemic immune systems, key beneficial traits to enhance development of the preterm gut and prevent NEC and late-onset sepsis.^25 28^ Furthermore, a multispecies approach may promote better colonisation because *Lactobacillus* may serve to reduce the oxygen content in the neonatal gastrointestinal tract, facilitating colonisation and persistence of anaerobic (ie, oxygen sensitive) *Bifidobacterium* species.^24^ However, it is critical to note that there are significant differences between individual *Bifidobacterium* and *Lactobacillus* species and also huge strain variability, not least in ability to digest dietary components such as HMOs and in their immune and infection modulatory traits. Such complexities may explain conflicting results of some previous RCTs, such as the large UK multicentre Probiotics in Preterm infants Study (the PiPS trial) which—using the single-strain probiotic *B*. *breve* BBG-001—did not reduce NEC or late-onset sepsis.^29^ Those findings may link to poor colonisation ability of the selected *B*. *breve* BBG-001 strain,^30^ compounded by low dosage used,^31^ and inability of this strain to digest key early life dietary components. *B*. *breve* strains frequently lack the genes required to assimilate HMO molecules, and consequently have limited ability to assimilate HMOs compared with *B*. *infantis* and *B*. *bifidum*.^32^ Further studies are needed to determine the generalisability of the underlying potential benefits of multispecies probiotics in this at-risk cohort.

## Conclusion

Administration of multispecies *Lactobacillus* and *Bifidobacterium* combination probiotics has been associated with a significantly decreased risk of NEC and late-onset sepsis in our NICU. This evaluation supports our routine use of multi-species *Lactobacillus* and *Bifidobacterium* combination probiotics for preventing NEC in very preterm neonates.

## References

[1] Battersby C, Longford N, Costeloe K, et al Development of a gestational age-specific case definition for neonatal necrotizing enterocolitis. JAMA Pediatr 2017;171:256–63. 10.1001/jamapediatrics.2016.3633 28046187

[2] Neu J, Pammi M Pathogenesis of NEC: impact of an altered intestinal microbiome. Semin Perinatol 2017;41:29–35. 10.1053/j.semperi.2016.09.015 27986328

[3] Duley L, Uhm S, Oliver S, et al Top 15 UK research priorities for preterm birth. The Lancet 2014;383:2041–2. 10.1016/S0140-6736(14)60989-2 24931684

[4] Gordon PV, Swanson JR, MacQueen BC, et al A critical question for NEC researchers: can we create a consensus definition of NEC that facilitates research progress? Semin Perinatol 2017;41:7–14. 10.1053/j.semperi.2016.09.013 27866661

[5] Berrington JE, Stewart CJ, Embleton ND, et al Gut microbiota in preterm infants: assessment and relevance to health and disease. Arch Dis Child Fetal Neonatal Ed 2013;98:F286–90. 10.1136/archdischild-2012-302134 23009761

[6] Hill C, Guarner F, Reid G, et al The International scientific association for probiotics and prebiotics consensus statement on the scope and appropriate use of the term probiotic. Nat Rev Gastroenterol Hepatol 2014;11:506–14. 10.1038/nrgastro.2014.66 24912386

[7] AlFaleh K, Anabrees J Probiotics for prevention of necrotizing enterocolitis in preterm infants. Cochrane Database Syst Rev 2014:CD005496. 24723255 10.1002/14651858.CD005496.pub4

[8] Olsen R, Greisen G, Schrøder M, et al Prophylactic probiotics for preterm infants: a systematic review and meta-analysis of observational studies. Neonatology 2016;109:105–12. 10.1159/000441274 26624488

[9] Dermyshi E, Wang Y, Yan C, et al The “Golden Age” of Probiotics: A Systematic Review and Meta-Analysis of Randomized and Observational Studies in Preterm Infants. Neonatology 2017;112:9–23. 10.1159/000454668 28196365

[10] Wang Q, Dong J, Zhu Y Probiotic supplement reduces risk of necrotizing enterocolitis and mortality in preterm very low-birth-weight infants: an updated meta-analysis of 20 randomized, controlled trials. J Pediatr Surg 2012;47:241–8. 10.1016/j.jpedsurg.2011.09.064 22244424

[11] Chang H-Y, Chen J-H, Chang J-H, et al Multiple strains probiotics appear to be the most effective probiotics in the prevention of necrotizing enterocolitis and mortality: an updated meta-analysis. PLoS One 2017;12:e0171579 10.1371/journal.pone.0171579 28182644 PMC5300201

[12] Duffield SD, Clarke P Current use of probiotics to prevent necrotising enterocolitis. Arch Dis Child Fetal Neonatal Ed 2019;104:F228 10.1136/archdischild-2018-316199 30464004

[13] van den Akker CHP, van Goudoever JB, Szajewska H, et al Probiotics for preterm infants: a strain-specific systematic review and network meta-analysis. J Pediatr Gastroenterol Nutr 2018;67:103–22. 29384838 10.1097/MPG.0000000000001897

[14] Deshpande GC, Rao SC, Keil AD, et al Evidence-Based guidelines for use of probiotics in preterm neonates. BMC Med 2011;9:92 10.1186/1741-7015-9-92 21806843 PMC3163616

[15] Sesham R, Oddie S, Embleton ND, et al Probiotics for preterm neonates: parents’ perspectives and present prevalence. Arch Dis Child Fetal Neonatal Ed 2014;99:F345–345. 10.1136/archdischild-2014-306344 24723695

[16] Alcon-Giner C, Dalby M, Caim S, et al Microbiota supplementation with Bifidobacterium and Lactobacillus modifies the preterm infant gut microbiota and metabolome. bioRxiv 2019;698092. 10.1016/j.xcrm.2020.100077 PMC7453906 32904427

[17] Brown E, Dabbour S, Clapuci R, et al 4 years' routine administration of probiotics to high-risk neonates: retrospective audit of adherence ot local guidelines. 7th Congress of the European Academy of Paediatrc Societies (EAPS) October 30-November 3,2018; Paris, France. Available: https://public-poster-links.s3.amazonaws.com/EAPS-2018/EAPS-2018-EAPS8-0834.pdf [Accessed 18 Oct 2019].

[18] Stenson B, Brocklehurst P, Tarnow-Mordi W, et al Increased 36-week survival with high oxygen saturation target in extremely preterm infants. N Engl J Med 2011;364:1680–2. 10.1056/NEJMc1101319 21524227

[19] Walsh MC, Kliegman RM Necrotizing enterocolitis: treatment based on staging criteria. Pediatr Clin North Am 1986;33:179–201. 10.1016/S0031-3955(16)34975-6 3081865 PMC7131118

[20] Vermont Oxford Network 2019 Manual of Operations, Part 2: Data Definitions & Infant Data Forms. Release 23.2, February 2019. Available: https://vtoxford.zendesk.com/hc/en-us/article_attachments/360024732954/Manual_of_Operations_Part_2_v23.2.pdf [Accessed 18 Oct 2019].

[21] Thistlethwaite DL, Campbell DT Regression-discontinuity analysis: an alternative to the ex post FACTo experiment. J Educ Psychol 1960;51:309–17. 10.1037/h0044319

[22] Denkel LA, Schwab F, Garten L, et al Protective effect of Dual-Strain probiotics in preterm infants: a multi-center time series analysis. PLoS One 2016;11:e0158136 10.1371/journal.pone.0158136 27332554 PMC4917100

[23] Houghteling PD, Walker WA Why is initial bacterial colonization of the intestine important to Infantsʼ and Childrenʼs health? J Pediatr Gastroenterol Nutr 2015;60:294–307. 10.1097/MPG.0000000000000597 25313849 PMC4340742

[24] Koskella B, Hall LJ, Metcalf CJE The microbiome beyond the horizon of ecological and evolutionary theory. Nat Ecol Evol 2017;1:1606–15. 10.1038/s41559-017-0340-2 29038487

[25] O'Neill I, Schofield Z, Hall LJ Exploring the role of the microbiota member Bifidobacterium in modulating immune-linked diseases. Emerging Topics in Life Sciences 2017;1:333–49. 10.1042/ETLS20170058 PMC7288987 33525778

[26] Frese SA, Hutton AA, Contreras LN, et al Persistence of Supplemented Bifidobacterium longum subsp. infantis EVC001 in Breastfed Infants. mSphere 2017;2:501–17. 10.1128/mSphere.00501-17 PMC5717325 29242832

[27] O'Callaghan A, van Sinderen D Bifidobacteria and their role as members of the human gut microbiota. Front Microbiol 2016;7:925 10.3389/fmicb.2016.00925 27379055 PMC4908950

[28] Fukuda S, Toh H, Taylor TD, et al Acetate-producing bifidobacteria protect the host from enteropathogenic infection via carbohydrate transporters. Gut Microbes 2012;3:449–54. 10.4161/gmic.21214 22825494

[29] Costeloe K, Hardy P, Juszczak E, et al Bifidobacterium breve BBG-001 in very preterm infants: a randomised controlled phase 3 trial. The Lancet 2016;387:649–60. 10.1016/S0140-6736(15)01027-2 26628328

[30] Millar M, Seale J, Greenland M, et al The microbiome of infants recruited to a randomised placebo-controlled probiotic trial (PIPs trial). EBioMedicine 2017;20:255–62. 10.1016/j.ebiom.2017.05.019 28571671 PMC5478240

[31] Deshpande G, Rao S, Athalye-Jape G, et al Probiotics in very preterm infants: the PIPs trial. The Lancet 2016;388 10.1016/S0140-6736(16)31271-5 27533430

[32] Gotoh A, Katoh T, Sakanaka M, et al Sharing of human milk oligosaccharides degradants within bifidobacterial communities in faecal cultures supplemented with Bifidobacterium bifidum. Sci Rep 2018;8:13958 10.1038/s41598-018-32080-3 30228375 PMC6143587

