# Supplementary material for: Incidence of necrotising enterocolitis before and after introducing routine prophylactic Lactobacillus and Bifidobacterium probiotics
Source: Arch Dis Child Fetal Neonatal Ed. 2019 Oct 30;105(4):380–6. doi: 10.1136/archdischild-2019-317346 (PMC7363787; doi:10.1136/archdischild-2019-317346)
Supplement: Supplementary data [file fetalneonatal-2019-317346supp001.pdf]

Online Supplementary File 1: Detail of statistical methods

Cox proportional hazards regression was used throughout to estimate the effect of epoch on the rates of NEC and all-cause mortality. Days since birth was used as time scale in primary analysis, with corrected gestational age used as time scale in a sensitivity analysis. Neonates were considered to be at risk of NEC from birth until the first of (i) death (ii) discharge home or (iii) censoring on 1 Jan 2013, if born before this date.

Death and discharge home were considered 'competing risks', as neonates are no longer at risk of NEC after either event. Competing risks were entered into the models using the approach of Fine and Gray.[1] All measured covariates were included in regression models. Enteral feed type was coded as a time-varying covariate, as 'no enteral feed' until the initiation of feeds (or until an event: NEC, discharge, censoring, or death) and thereafter categorised as one of the feed types listed above.

Neonates alive but with no NEC diagnosis and not yet discharged on 1 January 2013 were censored on that date.

Following the initial analysis, regression diagnostics showed that the proportional hazards assumption was strongly violated. Hence the model was re-estimated using time-varying covariate. Visual inspection of the cumulative incidence curve suggested that the effect of epoch was different in the risk periods of 'up to 1 week', '1-2 weeks', and '>2 weeks' and so we allowed separate estimates for the effect during each time.

Finally, the effect of time on NEC in each stratum was estimated using a regression discontinuity analysis, that is simultaneously including date of birth dichotomised into epochs as well as the effect of calendar year entered as a continuous linear covariate. This tests whether any observed differences are better explained by a linear time trend or by a step change after the change in probiotic-use policy.[2] For illustrative purposes, logistic

regression was used to estimate and subsequently plot the overall rate of NEC per admission and how this varied with the interaction of calendar year and epoch.

To estimate the effect on all-cause mortality, a second Cox regression was conducted with mortality as the index event, discharge home as a competing risk, and censoring at 1st January 2013. Since there was no effect of epoch on mortality after adjusting for covariates in this analysis we did not conduct the regression discontinuity analysis for mortality.

There were very few missing data for outcomes or most covariates; any cases with missing data in any analysis were dropped from that analysis, except for 'PROM' where more cases were missing and a separate 'not recorded' category was created.

All analysis was conducted using R statistical software (version 3.5.0) using the base statistics and 'survival' (version 2.43) package.[3] [4]

References:

[1] Fine JP, Gray RJ. A proportional hazards model for the subdistribution of a competing risk. *J Am Stat Assoc*. 1999;94:496-509.

[2] Thistlethwaite D, Campbell D. "Regression-Discontinuity Analysis: An alternative to the ex post facto experiment". *J Educ Psychol* 1960;51:309–17. doi:10.1037/h0044319.

[3] R Core Team (2018). R: A language and environment for statistical computing. R Foundation for Statistical Computing, Vienna, Austria. URL: https://www.R-project.org/.

[4] Therneau T (2015). *A Package for Survival Analysis in S*. version 2.38, https://CRAN.R-project.org/package=survival.
