# Supplementary figures and images for: Incidence of necrotising enterocolitis before and after introducing routine prophylactic Lactobacillus and Bifidobacterium probiotics
Source: Arch Dis Child Fetal Neonatal Ed. 2019 Oct 30;105(4):380–6. doi: 10.1136/archdischild-2019-317346 (PMC7363787; doi:10.1136/archdischild-2019-317346)

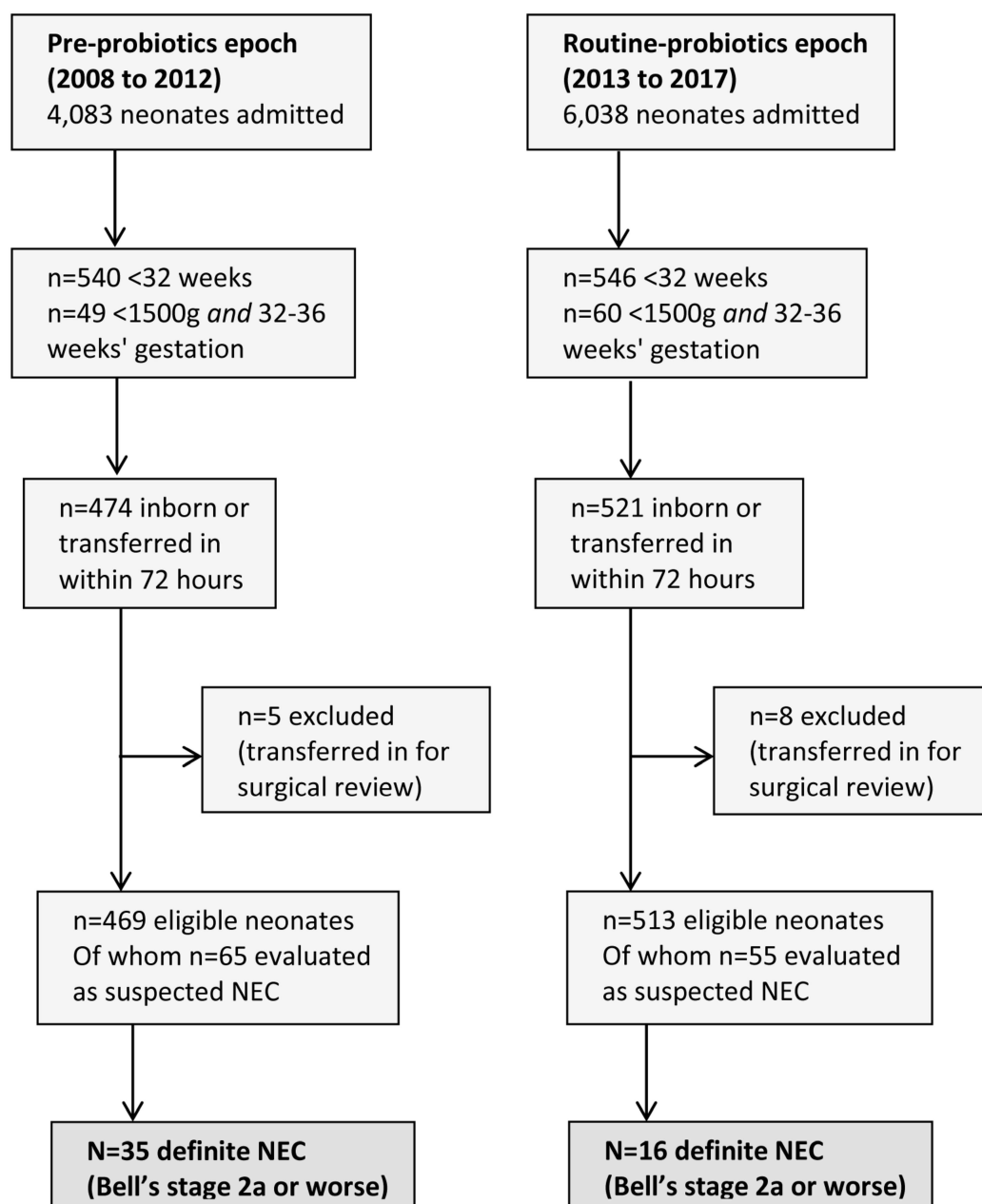

Online Supplementary File Figure S2: Patient flow in the pre- and routine-probiotics epochs

Supplement: Supplementary data [file fetalneonatal-2019-317346supp002.pdf]
