# Supplementary material for: Incidence of necrotising enterocolitis before and after introducing routine prophylactic Lactobacillus and Bifidobacterium probiotics
Source: Arch Dis Child Fetal Neonatal Ed. 2019 Oct 30;105(4):380–6. doi: 10.1136/archdischild-2019-317346 (PMC7363787; doi:10.1136/archdischild-2019-317346)
Supplement: Supplementary data [file fetalneonatal-2019-317346supp003.pdf]

Supplementary File Table S3. The association between the cumulative incidence of NEC, epoch and other predictors, estimated using three separate regression models. The first is a proportional hazards regression with a single parameter for the association between NEC and epoch. The second allows the association between NEC and epoch to vary with time since birth. The third is as the second but with the addition of a factor representing calendar year, thereby estimating and controlling for a linear trend with time.

		**Single predictor**		**Time-varying effect**		**Regression Discontinuity**	
Factor	*Level*	*Sub Hazard Ratio (95% CI)*	*p-value*	*Sub Hazard Ratio (95% CI)*	*p-value*	*Sub Hazard Ratio (95% CI)*	*p-value*
Epoch	Routine vs pre-probiotics	0.44 (0.23–0.85)	0.014	-	-	-	-
Epoch: first week after birth	Routine vs pre-probiotics	-	-	0.08 (0.01–0.59)	0.014	0.04 (0.00–0.63)	0.023
Epoch: second week after birth	Routine vs pre-probiotics	-	-	0.43 (0.13–1.42)	0.166	0.68 (0.07–6.74)	0.740
Epoch: beyond second week	Routine vs pre-probiotics	-	-	0.85 (0.36–2.01)	0.716	1.01 (0.18–5.67)	0.992
Calendar year: first week after birth	Per year	-	-	-	-	1.16 (0.77–1.75)	0.479
Calendar year: second week after birth	Per year	-	-	-	-	0.91 (0.62–1.36)	0.656
Calendar year: beyond second week	Per year	-	-	-	-	0.97 (0.72–1.30)	0.838
Gestational age at birth	<25 weeks	1.00 (Ref)		1.00 (Ref)		1.00 (Ref)	
	25-26 weeks	0.34 (0.16–0.70)	0.003	0.32 (0.16–0.66)	0.002	0.32 (0.16–0.67)	0.002
	27-30 weeks	0.09 (0.03–0.26)	<0.001	0.08 (0.03–0.25)	<0.001	0.09 (0.03–0.25)	<0.001
	31+ weeks						

Milk type	Mother's milk	1.00 (Ref)		1.00 (Ref)		1.00 (Ref)	
	No enteral feed	0.15 (0.04–0.52)	0.003	0.22 (0.06–0.87)	0.030	0.23 (0.06–0.91)	0.036
	Donor milk*	-	-	-	-	-	-
	Formula feed	0.85 (0.29–2.52)	0.775	0.82 (0.28–2.43)	0.724	0.85 (0.28–2.55)	0.768
	Mixed	0.82 (0.41–1.64)	0.576	0.80 (0.40–1.61)	0.533	0.81 (0.40–1.62)	0.549
Birthweight	<1000g	1.00 (Ref)		1.00 (Ref)		1.00 (Ref)	
	1000-1499g	0.71 (0.26–1.99)	0.520	0.70 (0.25–1.94)	0.491	0.70 (0.25–1.93)	0.487
	>1500g	1.12 (0.22–5.84)	0.891	1.12 (0.21–5.81)	0.896	1.11 (0.21–5.76)	0.904
Sex	Male (vs Female)	1.05 (0.59–1.86)	0.872	1.04 (0.58–1.84)	0.903	1.05 (0.59–1.87)	0.878
Antenatal steroids	At least one dose (vs never)	1.10 (0.45–2.68)	0.840	1.14 (0.47–2.80)	0.772	1.12 (0.46–2.76)	0.798
NSAID	None	1.00 (Ref)		1.00 (Ref)		1.00 (Ref)	
	Indometacin	1.21 (0.47–3.08)	0.694	1.14 (0.45–2.91)	0.786	1.16 (0.43–3.11)	0.768
	Ibuprofen	1.20 (0.41–3.56)	0.738	1.15 (0.39–3.43)	0.796	1.15 (0.39–3.41)	0.805
PROM	No	1.00 (Ref)		1.00 (Ref)		1.00 (Ref)	
	Yes	0.53 (0.22–1.29)	0.164	0.49 (0.20–1.20)	0.119	0.49 (0.20–1.19)	0.115
	Not recorded	0.78 (0.33–1.88)	0.583	0.78 (0.33–1.88)	0.584	0.77 (0.32–1.87)	0.568
Mode of Birth	Caesarean (vs vaginal)	1.82 (0.92–3.60)	0.086	1.72 (0.87–3.37)	0.116	1.72 (0.87–3.37)	0.118

* No baby received donor milk in the first epoch and there was no case of NEC among the infants (N=9) who were exclusively donor milk fed in the routine probiotics epoch.
