# Supplementary material for: Incidence of necrotising enterocolitis before and after introducing routine prophylactic Lactobacillus and Bifidobacterium probiotics
Source: Arch Dis Child Fetal Neonatal Ed. 2019 Oct 30;105(4):380–6. doi: 10.1136/archdischild-2019-317346 (PMC7363787; doi:10.1136/archdischild-2019-317346)
Supplement: Supplementary data [file fetalneonatal-2019-317346supp004.pdf]

Supplementary file table S4. Multivariable regression showing the sub-hazard ratios for death associated with each factor.

CI=confidence interval; IQR=inter-quartile range; PROM=premature rupture of membranes;

Factor	Level	Sub-Hazard Ratio (95%CI)	p-value
Epoch	Post vs pre-probiotics	0.74 (0.49–1.12)	0.155
Gestational age at birth	<25 weeks	1.00 (Ref)	
	25-26 weeks	0.28*** (0.17–0.47)	<0.001
	27-30 weeks	0.12*** (0.06–0.25)	<0.001
	31+ weeks	0.09*** (0.03–0.26)	<0.001
Milk type‡	Mother's milk	1.00 (Ref)	
	No enteral feed	2.24* (1.15–4.38)	0.018
	Donor milk	2.30 (0.31–17.09)	0.414
	Formula feed	0.35 (0.11–1.14)	0.082
	Mixed	0.51* (0.27–0.97)	0.039
Birthweight	<1000g	1.00 (Ref)	
	1000-1499g	0.53 (0.26–1.07)	0.076
	>1500g	0.68 (0.25–1.87)	0.460
Sex	Male (vs Female)	1.23 (0.84–1.80)	0.281
Antenatal steroids	At least once (vs never)	0.56* (0.35–0.90)	0.017
NSAID	None	1.00 (Ref)	
	Indometacin	1.28 (0.69–2.37)	0.434
	Ibuprofen	0.35* (0.12–0.97)	0.043
PROM	No	1.00 (Ref)	
	Yes	0.88 (0.54–1.43)	0.605
	Not recorded	0.93 (0.54–1.61)	0.799
Mode of Birth	Caesarean (vs vaginal)	1.07 (0.67–1.70)	0.790

NSAID= non-steroidal anti-inflammatory drug; Ref.=reference group.
‡Mode of milk feeding between first feed and full feeds or NEC/death if earlier
***=p<0.001, **=p<0.01; *=p<0.05.
