# Supplementary material for: Incidence of necrotising enterocolitis before and after introducing routine prophylactic Lactobacillus and Bifidobacterium probiotics
Source: Arch Dis Child Fetal Neonatal Ed. 2019 Oct 30;105(4):380–6. doi: 10.1136/archdischild-2019-317346 (PMC7363787; doi:10.1136/archdischild-2019-317346)
Supplement: Supplementary data [file fetalneonatal-2019-317346supp005.pdf]

Supplementary File Table S5. The association between death, epoch and other predictors, estimated using three separate regression models. The first is a proportional hazards regression with a single parameter for the association between death and epoch. The second allows the association between death and epoch to vary with time since birth. The third is as the second but with the addition of a factor representing calendar year, thereby estimating and controlling for a linear trend with time.

		**'Epoch' as a single predictor**		**Time-varying effect**		**Regression Discontinuity**	
Level	*Level*	*Sub Hazard Ratio (95% CI)*	*p-value*	*Sub Hazard Ratio(95% CI)*	*p-value*	*Sub Hazard Ratio(95% CI)*	*p-value*
Epoch	Routine vs pre-probiotics	0.74 (0.49–1.12)	0.155				
Epoch: first week after birth	Routine vs pre-probiotics			1.24 (0.72–2.15)	0.434	0.78 (0.26–2.36)	0.665
Epoch: second week after birth	Routine vs pre-probiotics			0.74 (0.26–2.12)	0.579	0.58 (0.07–4.92)	0.620
Epoch: beyond second week	Routine vs pre-probiotics			0.34 (0.17–0.68)	0.003	2.05 (0.52–8.09)	0.305
Calendar year: first week after birth	Per year					1.09 (0.90–1.31)	0.380
Calendar year: second week after birth	Per year					1.04 (0.74–1.47)	0.829
Calendar year: beyond second week	Per year					0.70 (0.55–0.88)	0.003
Gestational age at birth	<25 weeks	1.00 (Ref)		1.00 (Ref)		1.00 (Ref)	
	25-26 weeks	0.28 (0.17–0.47)	<0.001	0.27 (0.17–0.45)	<0.001	0.27 (0.16–0.44)	<0.001
	27-30 weeks	0.12 (0.06–0.25)	<0.001	0.12 (0.06–0.25)	<0.001	0.12 (0.06–0.24)	<0.001
	31+ weeks	0.09 (0.03–0.26)	<0.001	0.09 (0.03–0.26)	<0.001	0.09 (0.03–0.26)	<0.001

Milk type	Mother's milk	1.00 (Ref)		1.00 (Ref)		1.00 (Ref)	
	No enteral feed	2.24 (1.15–4.38)	0.018	2.28 (1.18–4.43)	0.015	2.28 (1.18–4.41)	0.014
	Donor milk	2.30 (0.31–17.09)	0.414	3.08 (0.41–23.25)	0.275	3.00 (0.40–22.73)	0.288
	Formula feed	0.35 (0.11–1.14)	0.082	0.31 (0.09–1.01)	0.053	0.27 (0.08–0.89)	0.031
	Mixed	0.51 (0.27–0.97)	0.039	0.45 (0.24–0.86)	0.016	0.40 (0.21–0.77)	0.006
Birthweight	<1000g	1.00 (Ref)		1.00 (Ref)		1.00 (Ref)	
	1000-1499g	0.53 (0.26–1.07)	0.076	0.53 (0.27–1.07)	0.077	0.53 (0.27–1.07)	0.075
	>1500g	0.68 (0.25–1.87)	0.460	0.68 (0.25–1.87)	0.456	0.69 (0.25–1.89)	0.467
Sex	Male (vs Female)	1.23 (0.84–1.80)	0.281	1.22 (0.84–1.79)	0.296	1.20 (0.82–1.75)	0.358
Antenatal steroids	At least one dose (vs never)	0.56 (0.35–0.90)	0.017	0.55 (0.34–0.88)	0.013	0.56 (0.35–0.91)	0.019
NSAID	None	1.00 (Ref)		1.00 (Ref)		1.00 (Ref)	
	Indometacin	1.28 (0.69–2.37)	0.434	1.31 (0.71–2.43)	0.388	1.18 (0.62–2.25)	0.613
	Ibuprofen	0.35 (0.12–0.97)	0.043	0.35 (0.13–0.97)	0.044	0.34 (0.12–0.94)	0.038
PROM	No	1.00 (Ref)		1.00 (Ref)		1.00 (Ref)	
	Yes	0.88 (0.54–1.43)	0.605	0.89 (0.54–1.45)	0.628	0.91 (0.56–1.48)	0.695
	Not recorded	0.93 (0.54–1.61)	0.799	0.94 (0.54–1.62)	0.816	0.91 (0.52–1.58)	0.734
Mode of Birth	Caesarean (vs vaginal)	1.07 (0.67–1.70)	0.790	1.06 (0.67–1.70)	0.797	1.08 (0.68–1.72)	0.749
